# Implementation and Evaluation of a Pilot Narrative Medicine Curriculum for Internal Medicine Residents

**DOI:** 10.7759/cureus.53396

**Published:** 2024-02-01

**Authors:** Rebecca K Tsevat, Peter Young, Eunice Zhang, Samuel Baugh, Antonio M Pessegueiro

**Affiliations:** 1 Department of Medicine, Division of General Internal Medicine and Health Services Research, University of California, Los Angeles David Geffen School of Medicine, Los Angeles, USA; 2 Department of Medicine, Division of Internal Medicine-Pediatrics, University of California, Los Angeles David Geffen School of Medicine, Los Angeles, USA; 3 National Clinician Scholars Program, University of California, Los Angeles, Los Angeles, USA; 4 Department of Medicine, West Los Angeles Veterans Affairs Medical Center, Los Angeles, USA; 5 Department of Statistics, Penn State University, University Park, USA; 6 Department of Statistics, University of California, Los Angeles, Los Angeles, USA

**Keywords:** graduate medical education, med-peds, internal medicine, medical humanities, narrative medicine

## Abstract

Background

Narrative medicine has been integrated into medical training to enhance competencies such as observation, reflection, and self-care. However, few studies have assessed the impact of a single narrative medicine session using a pre- and post-test study design. The authors of this study sought to implement a pilot narrative medicine curriculum into a large internal medicine residency program and to evaluate its feasibility and impact.

Methodology

The curriculum consisted of a one-hour reading and writing workshop held during ambulatory academic half-days from 2021 to 2022. Resident participants completed a retrospective pre- and post-workshop survey evaluating their interest and confidence in practicing narrative medicine skills, as well as their beliefs about the impacts of narrative medicine on patient care and provider well-being. Descriptive statistics evaluated pre- and post-workshop differences using the Wilcoxon signed-rank test. Subgroup analyses were conducted based on postgraduate year, residency track, and workshop setting. Additionally, participants completed open-ended questions that were analyzed qualitatively.

Results

Of 218 resident participants, 152 (69.7%) completed the post-session survey. Participants noted significantly higher levels of confidence and interest in listening to patient stories, analyzing literary texts, and engaging in reflective writing after the workshop. They also expressed significantly higher levels of agreement that engaging in literary analysis and reflective writing could improve patient care, reduce provider burnout, and strengthen connectedness with colleagues. Qualitative analysis demonstrated that participants found the sessions to be worthwhile and appreciated how narrative medicine could enhance their medical practice.

Conclusions

Incorporating a brief narrative medicine curriculum into an internal medicine residency program is both feasible and valuable. A single narrative medicine session was practical and well-received by residents, as it promoted self-reflection, observational skills, and connection with colleagues. Future workshops should be customized for different training levels and residency tracks, and additional studies should evaluate whether the outcomes persist over time.

## Introduction

Narrative medicine is defined as “medicine practiced with the skills of recognizing, absorbing, interpreting, and being moved by stories of illness” [1]. Listening to and telling stories are key skills for every clinician, and narrative skills enable one person to receive and understand the stories told by another. Studies have found that incorporating narrative medicine into clinical education is linked to increased empathy, communication, observation, and ethical reasoning skills [2]. Additionally, narrative medicine curricula have been shown to promote peer-to-peer learning and affiliation [3], enhance personal growth and identity formation [4], and enable reflection and self-care [5]. Individual studies have also shown improvements in measures of cultural competency [6], resiliency and burnout [7-9], and professionalism [10] as a result of exposure to narrative medicine training.

Narrative medicine has been integrated into medical training with increased frequency over the past several years. Remein et al. conducted a systematic review of 55 narrative medicine programs in healthcare settings, the majority of which were offered to medical trainees [11]. A core element of narrative medicine practice is the workshop, which involves close reading of a literary text followed by discussion and prompted writing [12]; all of the studies included in the systematic review featured textual analysis or close reading of published literature as well as creative or reflective writing. However, the programs varied widely in terms of number of sessions, number of participants, and method of program evaluation. The vast majority featured multiple sessions, had a limited number of participants, and used a cross-sectional study design with only a post-test and no pre-test [11]. Significant outcomes included improvements in communication and team-building skills, perspective-taking and reflection, empathic behavior, detection and mitigation of burnout, narrative competence, pedagogical and clinical skills, and ethical inquiry [11].

Narrative medicine curricula designed specifically for resident physicians have previously been described in the literature and span several disciplines, including obstetrics and gynecology, pediatrics, surgery, and internal medicine [9,13-15]. Nevertheless, few studies have described the impact of a single narrative medicine session on a large number of resident participants using a pre- and post-test study design. The advantages of this approach include opportunities to optimize flexibility in scheduling, broaden the scope of impact, and compare outcomes before and after the intervention. Accordingly, this pilot educational study aimed to develop a narrative medicine curriculum consisting of a single workshop for all internal medicine residents at the authors’ institution, as well as to evaluate several resident-centered outcomes. The authors hypothesized that residents would feel more confident and interested in practicing skills relevant to narrative medicine, as well as attribute greater value to their utility and applicability to patient care, after participating in the intervention.

The results of this study were previously presented in a poster at the Society of General Internal Medicine (SGIM) Annual Meeting in Orlando, Florida on April 7, 2022, and in an oral presentation at the virtual SGIM Northwest & California-Hawaii Regional Meeting on January 21, 2022.

## Materials and methods

This narrative medicine intervention consisted of a one-hour interactive workshop for multiple small groups of residents. All workshops occurred in the mornings during ambulatory academic half-days between January 2021 and February 2022, with each session consisting of residents from one postgraduate year. Twenty-two workshops were conducted in total: twenty for categorical track residents and two for primary care track and med-peds residents. Each workshop was attended by 8-16 residents, who completed a single session during the study period. Due to the COVID-19 pandemic, 12 workshops were conducted online over Zoom with the remainder held in person.

The workshops began with a brief introduction to the field of narrative medicine followed by a reading and discussion of one of two poems: “The Ship Pounding” by Donald Hall [16] and “It was Already Dangerous” by Lauren Whitehead [17]. These poems were selected because of their brief length, perceived accessibility, and thematic focus on illness (Appendix 1: Choosing a Text and Prompt). Participants were prompted to attend to the literary elements of these poems, focusing on their structural and linguistic details, and to share their observations aloud (Appendix 2: Workshop Teaching Guides). Following this discussion, one of two writing prompts was presented, each designed to encourage personal reflection and perspective-taking. For those who read “The Ship Pounding,” the prompt was to “write in the voice of a patient or their family member.” For those who read “It Was Already Dangerous,” the prompt was to “write about the invisible labor of someone you know.” Participants were asked to write for five minutes and then invited to share their writing with the group, who would respond with comments and reflection.

Following the workshop, participants completed a retrospective pre- and post-survey assessing their interest and confidence in practicing narrative medicine skills, as well as their beliefs about the impacts of narrative medicine on patient care and provider well-being (Appendix 3: Survey Questions). The survey was homegrown with no collection of validity evidence. All questions consisted of a five-point Likert scale, with higher numbers representing higher values. Pre- and post-workshop differences were analyzed using the Wilcoxon signed-rank test for non-parametric ordinal response variables; statistical significance was considered at p-values <0.05. Subgroup analyses were conducted based on postgraduate year, residency track, and workshop setting. Additionally, participants completed open-ended questions assessing what they learned from the session and what they could apply to their future practice. The participants’ responses were analyzed using an iterative process, including open coding, to identify emergent themes. The survey was administered through a HIPAA-compliant online platform, and no personally identifiable data were collected. This educational study was reviewed by the institutional review board office and was certified exempt as it did not meet the institution’s definition of human subjects research.

## Results

Participants

All participants were internal medicine residents at the authors’ institution. The study sample consisted of 64 interns, 63 second-year residents, and 91 third-year residents. The majority (186) were categorical residents, and the remainder (32) were primary care track or med-peds residents. Of 218 residents who attended the workshops, 152 (69.7%) completed the post-session survey.

Quantitative

Participants’ level of interest in learning about narrative medicine, analyzing literature, and engaging in reflective writing increased significantly after the workshop compared to prior (p < 0.001 for all comparisons). Additionally, participants noted significantly higher confidence in their ability to listen to patient stories, analyze literature, and engage in reflective writing (p < 0.001 for all comparisons). They also expressed higher levels of agreement with the notions that engaging in literary analysis and reflective writing could improve patient care, reduce provider burnout, and improve connectedness to colleagues (p < 0.001 for all comparisons) (Table 1).

**Table 1:** Mean difference in scores among all participants (N = 152). ^1^: Rated on a five-point Likert scale (1 = strongly disagree, 5 = strongly agree).

Survey topic	Mean pre-workshop score^1^	Mean post-workshop score^1^	Mean difference	P-value
Confidence in listening to patients’ stories	3.08	3.62	0.54	<0.001
Confidence in analyzing literature	2.62	3.30	0.68	<0.001
Confidence in reflective writing	2.29	3.08	0.79	<0.001
Interest in learning about narrative medicine	3.18	3.69	0.51	<0.001
Interest in analyzing literature	2.99	3.57	0.58	<0.001
Interest in reflective writing	2.90	3.54	0.64	<0.001
Agreement that literary analysis and reflective writing can improve patient care	3.88	4.36	0.48	<0.001
Agreement that literary analysis and reflective writing can reduce burnout	4.01	4.43	0.42	<0.001
Agreement that literary analysis and reflective writing can improve connectedness with colleagues	4.14	4.50	0.36	<0.001

Subgroup analyses were conducted based on postgraduate year, residency track, and workshop setting. Among interns, levels of confidence in listening to patient stories (p = 0.01), analyzing literature (p < 0.001), and engaging in reflective writing (p < 0.001) increased after the workshop. Additionally, interns expressed greater agreement that engaging in literary analysis and reflective writing could improve burnout (p = 0.02) but not patient care (p = 0.09) or connectedness to colleagues (p = 1.00). After the workshop, second-year residents reported more confidence in analyzing literature (p = 0.004) and engaging in reflective writing (p < 0.001) but not in listening to patient stories (p = 0.10). They also expressed greater agreement that engaging in literary analysis and reflective writing could improve patient care (p = 0.02), but this difference did not extend to burnout (p = 0.13) or connectedness to colleagues (p = 0.13). For third-year residents, there were statistically significant differences across all survey questions (Table 2).

**Table 2:** Mean difference in scores by postgraduate year (PGY) level. ^1^: Rated on a five-point Likert scale (1 = strongly disagree, 5 = strongly agree).

Survey topic	PGY level	Mean pre-workshop score^1^	Mean post-workshop score^1^	Mean difference	P-value
Confidence in listening to patients’ stories	1	3.15	3.77	0.62	0.01
Confidence in listening to patients’ stories	2	3.20	3.60	0.40	0.10
Confidence in listening to patients’ stories	3	2.93	3.53	0.60	<0.001
Confidence in analyzing literature	1	2.51	3.44	0.93	<0.001
Confidence in analyzing literature	2	2.87	3.49	0.62	0.004
Confidence in analyzing literature	3	2.49	3.05	0.56	<0.001
Confidence in reflective writing	1	2.18	3.10	0.92	<0.001
Confidence in reflective writing	2	2.51	3.18	0.67	<0.001
Confidence in reflective writing	3	2.19	2.98	0.79	<0.001
Interest in learning about narrative medicine	1	3.26	3.72	0.46	0.01
Interest in learning about narrative medicine	2	3.20	3.73	0.53	0.01
Interest in learning about narrative medicine	3	3.12	3.63	0.51	<0.001
Interest in analyzing literature	1	2.62	3.46	0.84	<0.001
Interest in analyzing literature	2	3.22	3.71	0.49	0.06
Interest in analyzing literature	3	3.05	3.54	0.49	0.002
Interest in reflective writing	1	2.77	3.41	0.64	0.01
Interest in reflective writing	2	3.09	3.73	0.64	<0.001
Interest in reflective writing	3	2.84	3.47	0.63	<0.001
Agreement that literary analysis and reflective writing can improve patient care	1	3.90	4.33	0.43	0.09
Agreement that literary analysis and reflective writing can improve patient care	2	3.96	4.40	0.44	0.02
Agreement that literary analysis and reflective writing can improve patient care	3	3.81	4.33	0.52	0.002
Agreement that literary analysis and reflective writing can reduce burnout	1	3.74	4.31	0.57	0.02
Agreement that literary analysis and reflective writing can reduce burnout	2	4.20	4.51	0.31	0.13
Agreement that literary analysis and reflective writing can reduce burnout	3	4.04	4.44	0.40	0.01
Agreement that literary analysis and reflective writing can improve connectedness with colleagues	1	4.21	4.49	0.28	1.00
Agreement that literary analysis and reflective writing can improve connectedness with colleagues	2	4.20	4.51	0.31	0.13
Agreement that literary analysis and reflective writing can improve connectedness with colleagues	3	4.04	4.51	0.47	0.001

After the workshop, primary care and med-peds residents reported greater confidence in listening to patients’ stories (p = 0.02), analyzing literature (p = 0.001), and engaging in reflective writing (p = 0.002), as well as greater interest in analyzing literature (p = 0.04) and engaging in reflective writing (p = 0.02). There were no significant differences in their interest in learning about narrative medicine (p = 0.22) or their agreement that engaging in literary analysis and reflective writing could improve patient care (p = 0.05), reduce burnout (p = 0.10), or improve connectedness to colleagues (p = 0.93) after the workshop. In contrast, categorial residents reported statistically significant differences across all questions (Table 3).

**Table 3:** Mean difference in scores by residency track (categorical vs. primary care or med-peds). ^1^: Rated on a five-point Likert scale (1 = strongly disagree, 5 = strongly agree).

Survey topic	Categorical (Y/N)	Mean pre-workshop score^1^	Mean post-workshop score^1^	Mean difference	P-value
Confidence in listening to patients’ stories	N	3.17	3.83	0.66	0.02
Confidence in listening to patients’ stories	Y	3.06	3.57	0.51	<0.001
Confidence in analyzing literature	N	2.54	3.58	1.04	0.001
Confidence in analyzing literature	Y	2.63	3.24	0.61	<0.001
Confidence in reflective writing	N	2.13	3.38	1.25	0.002
Confidence in reflective writing	Y	2.33	3.02	0.69	<0.001
Interest in learning about narrative medicine	N	3.50	4.04	0.54	0.22
Interest in learning about narrative medicine	Y	3.12	3.62	0.50	<0.001
Interest in analyzing literature	N	2.71	3.63	0.92	0.04
Interest in analyzing literature	Y	3.04	3.56	0.52	<0.001
Interest in reflective writing	N	2.96	3.96	1.00	0.02
Interest in reflective writing	Y	2.89	3.45	0.56	<0.001
Agreement that literary analysis and reflective writing can improve patient care	N	3.75	4.46	0.71	0.05
Agreement that literary analysis and reflective writing can improve patient care	Y	3.91	4.33	0.42	<0.001
Agreement that literary analysis and reflective writing can reduce burnout	N	3.96	4.54	0.58	0.10
Agreement that literary analysis and reflective writing can reduce burnout	Y	4.02	4.40	0.38	<0.001
Agreement that literary analysis and reflective writing can improve connectedness with colleagues	N	4.29	4.63	0.34	0.93
Agreement that literary analysis and reflective writing can improve connectedness with colleagues	Y	4.10	4.48	0.38	<0.001

Finally, 12 workshops were conducted online due to the COVID-19 pandemic. When comparing the responses of participants who attended the workshops in person versus over Zoom, both groups noted significant differences across all survey domains (Table 4).

**Table 4:** Mean difference in scores by workshop setting (in person vs. virtual). ^1^: Rated on a five-point Likert scale (1 = strongly disagree, 5 = strongly agree).

Survey topic	In person (Y/N)	Mean pre-workshop score^1^	Mean post-workshop score^1^	Mean difference	P-value
Confidence in listening to patients’ stories	N	2.93	3.58	0.65	<0.001
Confidence in listening to patients’ stories	Y	3.23	3.66	0.43	0.002
Confidence in analyzing literature	N	2.51	3.17	0.66	<0.001
Confidence in analyzing literature	Y	2.73	3.43	0.70	<0.001
Confidence in reflective writing	N	2.23	3.06	0.83	<0.001
Confidence in reflective writing	Y	2.36	3.10	0.74	<0.001
Interest in learning about narrative medicine	N	3.14	3.68	0.54	<0.001
Interest in learning about narrative medicine	Y	3.23	3.70	0.47	<0.001
Interest in analyzing literature	N	3.04	3.59	0.55	<0.001
Interest in analyzing literature	Y	2.93	3.56	0.63	<0.001
Interest in reflective writing	N	2.86	3.52	0.66	<0.001
Interest in reflective writing	Y	2.94	3.56	0.62	<0.001
Agreement that literary analysis and reflective writing can improve patient care	N	3.83	4.41	0.58	<0.001
Agreement that literary analysis and reflective writing can improve patient care	Y	3.93	4.30	0.37	0.003
Agreement that literary analysis and reflective writing can reduce burnout	N	4.01	4.48	0.47	<0.001
Agreement that literary analysis and reflective writing can reduce burnout	Y	4.00	4.37	0.37	0.01
Agreement that literary analysis and reflective writing can improve connectedness with colleagues	N	4.11	4.55	0.44	<0.001
Agreement that literary analysis and reflective writing can improve connectedness with colleagues	Y	4.16	4.46	0.30	0.04

Qualitative

Analysis of open-ended questions demonstrated that participants found the sessions to be engaging and worthwhile and that there were many skills that they could apply to their future practice. Four key themes were identified and centered around provider burnout, self-reflection, social connection, and patient care.

Some responses revealed that workshop participation directly impacted the level of burnout that had developed during residency, including during the COVID-19 pandemic:

“Reflective writing is very cathartic and really helps rediscover our humanism as doctors when work has beaten us down.”

“After completing this session, I feel like I have life in me again. Medicine has essentially ruined my perception of myself and being able to heal through even writing a 5-minute piece is amazing.”

“Being challenged to write in the voice of the patient, I think, was a helpful exercise in empathy building, even when facing pandemic burnout.”

Other responses demonstrated that the sessions promoted self-reflection and revealed what participants had learned about themselves:

“I have non-medical thoughts! This is important! I would like to embrace these aspects of myself more.”

“That I enjoyed writing and missed writing.”

“This was an act in 3D reflection. I was able to view myself as the patient, the provider, and as an outsider.”

Many responses reflected how the workshops fostered a connection with colleagues through the identification of shared experiences:

“Learned how similar a lot of our experiences are and how thoughtful and amazing my colleagues and friends are.”

“Impressed with how insightful and introspective my colleagues are! Reframing our work into a narrative form really shifts the perspective and gave me the sense of leaving ‘a routine’ or ‘a grind.’”

“I learned that my experience and perspective is shared by many of my colleagues, working long hours in the hospital with sick patients.”

Residents also revealed lessons related to patient care as they reflected on ways that narrative medicine could be applied to their clinical practice:

“I was so focused on filling out the basic H&P and I lost the narrative. [Would] like to be better listener and give patients the time to share their story.”

“I really struggled to think about hospitalization from a patient[‘s] perspective, therefore, this was helpful to ground me in what my patients may be feeling.”

“I definitely want to talk to patients more in my practice.”

## Discussion

The results of this pilot study suggest that incorporating a brief narrative medicine curriculum into an internal medicine residency program is both valuable and feasible. Participation in the sessions fostered meaningful reflection, development of observational skills, and connection with colleagues. Furthermore, a single, one-hour session was easily integrated into a pre-existing ambulatory academic half-day. There were few differences in responses between in-person and online participants, indicating that the workshop setting can allow for program flexibility. Additionally, the outcomes largely persisted across subgroups. While there were minor differences noted according to training level and residency track, post-workshop survey responses yielded widespread and significant gains in confidence, interest, and value assigned to narrative medicine skills among participants. These skills are especially important because they have implications for patient care and align with the Accreditation Council for Graduate Medical Education’s core competencies of Interpersonal and Communication Skills and Professionalism [18].

We learned several important lessons that will guide the next steps for this narrative medicine curriculum. Choosing a text that is relevant and accessible to each group is crucial, as a poem that works well for interns may not yield as valuable of a session for more advanced residents. Additionally, as responses to the workshops differed between cohorts, customization is critical to promote optimal engagement. In the future, we plan to tailor our sessions to a different theme each year: intern workshops will be related to patient experiences, second-year workshops to professional identity, and third-year workshops to burnout and disillusionment. Likewise, facilitators must be willing to adapt sessions to the needs of a particular group. Each group of residents may have a different level of enthusiasm and comfort with narrative medicine; forcing a group of reticent participants to engage beyond their comfort level can be counterproductive. Instead, it can be helpful to adjust the structure of the workshop by asking residents to share in pairs, for example, or to reflect on what they experienced during the writing process if they prefer not to disclose their written responses. In addition to customizing workshops to residents in different years of training and to groups with varying levels of comfort and familiarity with narrative medicine, future directions may include expanding the workshops to a broader audience and integrating them into pre-existing curricula that address issues such as Social Determinants of Health and Equity, Diversity, and Inclusion.

One limitation of our design is the need for a moderator. Most sessions were facilitated by one or two moderators who not only developed the curriculum but also had an intimate understanding of the principles of narrative medicine as well as experience in facilitating group sessions. A few sessions were led by chief residents with an interest in narrative medicine but less familiarity with the curriculum and facilitating experience. Another institution looking to adopt this model may find that their faculty leads have differing approaches and degrees of comfort when moderating, which can subsequently affect the discussion and insights generated. Regarding the workshop evaluation, limitations included using a single survey to evaluate pre- and post-workshop metrics, which may be subject to bias, and evaluating only short-term outcomes of the curriculum. Nevertheless, the retrospective pre- and post-survey administered directly after the workshop had the advantage of optimizing completion rates for a captive audience of participants. Future studies may build upon these limitations by using different evaluations before and after the workshop and evaluating whether the outcomes persist over time.

## Conclusions

This educational pilot study demonstrates that a brief narrative medicine curriculum can lead to improvements in self-reflective and observational skills among internal medicine residents. In addition to promoting resident well-being and connection with colleagues, these gains are important because they can impact the clinical care that residents deliver. Additionally, this study reveals that the single workshop design can be implemented either virtually or in person across multiple residency tracks and training levels. The study design, results, and lessons learned can inform other institutions seeking to implement practical and effective narrative medicine curricula into their residency programs. It will be important for educators to continue to evaluate the short- and long-term impacts of these curricula to optimize resident engagement and patient care, in turn.

## Appendices

Appendix 1: choosing a text and prompt

Choosing a text for a narrative medicine workshop

·      Choose a piece that addresses content or themes that will be relevant and evocative for a given group of residents. This is likely to vary based on the level of training and other factors, including current events and the timing of the workshop during clinical rotations.

·      The poem or passage should be one to two pages in length as it will be read twice during the session.

·      For groups new to narrative medicine, it may be helpful to choose a piece with content that is at least obliquely related to a medical narrative or experience. Nevertheless, care should be taken to emphasize that the goal is to focus not on content, but rather on how the words and structure achieve their impact.

·      A successful text should stimulate interesting discussion even after one to two readings given the nature of the workshop. It should also reward repeated readings and examinations.

·      The text should be inclusive in scope, language, and intended audience.

·      The material may, and perhaps should, be challenging, but moderators should be cautious with content that runs the risk of triggering its readers.

·      For repeated workshops with different groups, we recommend varying the texts to keep moderators engaged.

Choosing a prompt for a narrative medicine workshop

·      When creating a prompt, using specific language or themes from the text is often a good place to start.

·      A good prompt should lead to writing that often feels in conversation with the text.

·      The prompt should be short, clear, and easily understood, but also evocative, ambiguous, and expansive.

Appendix 2: workshop teaching guides

Workshop 1

Introduction

·      Personal introductions and icebreakers: name a piece of art (e.g., a book, film, or visual piece) that you have enjoyed recently.

Reading

·      Ask a volunteer to read “The Ship Pounding” by Donald Hall (full text below).

·      Ask a second volunteer to read, this time inviting residents to actively listen and take notes on interesting language and aspects of the poem that they would like to discuss.

Sample Discussion Questions

·      What are your initial thoughts after reading this poem?

·      What language do you find striking or surprising?

·      What does the poem make you see? Do certain images stand out to you?

·      How does time work in this poem? Are there any moments of transition that seem important?

·      What is the effect of the metaphor of the ship? Does it resonate?

·      How do you feel as a physician reading this poem?

Writing Prompt

·      Ask participants to write for 5 minutes in any format to the following prompt.

·      Prompt: “Write in the voice of a patient or their family member.”

Sharing of Reflective Writing

·      Ask for volunteers to share some or all of what they have written. Suggest that they share their writing without a preface.

·      Thank residents for sharing and ask colleagues to comment on what they hear. Encourage them to respond to the text and what they hear in the writing, rather than the content or the author’s intent. Remind them that it should be a safe, protected space for sharing.

·      If the group is reticent in sharing, ask them to comment on their experience of writing to the prompt.

Poem: “The Ship Pounding” by Donald Hall

Each morning I made my way

among gangways, elevators,

and nurses’ pods to Jane’s room

to interrogate the grave helpers

who tended her through the night

while the ship’s massive engines

kept its propellers turning.

Week after week, I sat by her bed

with black coffee and the Globe.

The passengers on this voyage

wore masks or cannulae

or dangled devices that dripped

chemicals into their wrists.

I believed that the ship

traveled to a harbor

of breakfast, work, and love.

I wrote: “When the infusions

are infused entirely, bone

marrow restored and lymphoblasts

remitted, I will take my wife,

bald as Michael Jordan,

back to our dog and day.” Today,

months later at home, these

words turned up on my desk

as I listened in case Jane called

for help, or spoke in delirium,

ready to make the agitated

drive to Emergency again

for readmission to the huge

vessel that heaves water month

after month, without leaving

port, without moving a knot,

without arrival or destination,

its great engines pounding.

Workshop 2

Introduction

·      Personal introductions and icebreakers: tell us something you have done recently that has brought you joy.

Reading

·      Ask a volunteer to read “It Was Already Dangerous,” by Lauren Whitehead (full text below).

·      Ask a second volunteer to read, this time inviting residents to actively listen and take notes on interesting language and aspects of the poem that they would like to discuss.

Sample Discussion Questions

·      Invite residents to speak about what they saw, heard, or felt in the text.

·      What language do you find striking or surprising?

·      What do you take the poem to be about? What feeling or mood is evoked, and how does the writer achieve this?

·      How did it feel reading the poem? What do you think about the pacing and the grammatical structure?

·      What does it mean to “bring [your] work home?”

·      Can you identify with any perspectives in this poem? If so, how does the poem invite identification? If not, why not?

Writing Prompt

·      Ask participants to write for 5 minutes in any format to the following prompt.

·      Prompt: “Write about the invisible labor of someone you know.”

Sharing of Reflective Writing

·      Ask for volunteers to share some or all of what they have written. Suggest that they share their writing without a preface.

·      Thank residents for sharing and ask colleagues to comment on what they hear. Encourage them to respond to the text and what they hear in the writing, rather than the content or the author’s intent. Remind them that it should be a safe, protected space for sharing.

·      If the group is reticent in sharing, ask them to comment on their experience of writing to the prompt. 

Poem: “It Was Already Dangerous” by Lauren Whitehead

Working the 2-12 shift Driving home in the shiny dark

under the sleepless moon Curling his car around

suburban back roads Almost every day, pushing

drowsily his nice-enough-to-not-get-pulled-over SUV

Iced coffee sugared and milked into cake It was

already dangerous, diabetic as he is, for him to be

smoking all these cigarettes in the empty parking lot,

laughing and missing all these meals, even

while working the 2-12 a.m. shift at the high-end grocery

where the cured meats have their own specified domain

Hanging hocks of pork sliced thin by a woman

in starched whites and a paper hat The grocery

where you build your own six-pack and also where

my dad manages young undereducated smokers

in the business of facing groceries as they come

out of the box You probably haven’t wondered

whose hands make known the difference between

scented and unscented garbage bags, which hands

attend the 200-plus flavors of tea in aisle three

of your local You probably pass, un-asking,

by the perfected symmetry of toothpastes

and soaps neatly packed, straight-backed like soldiers

But it’s my dad, working the near-night shift,

stacking organic frozen pizzas in the cooler, label out

so you don’t mistake your vegan for your four-cheese

He is a connoisseur of cabbage, a kale-fluffing man

who knows each condiment by its color-coded brand

And it was already laborious, throwing box after box

off a forklift, hauling pallets of pesto and pasta sauce

It was already heavy but now also all the extra loads

of alcohol, ammonia, bleach, dual action disinfectant

wipes & toilet paper all the near night, canned meats

and hard cheese and frozen everything He’s already 63,

the ideal vintage for an otherwise indiscriminate virus

which lives for days maybe on hard surfaces like

linoleum grocery floors or metal grocery racks or

aluminum soup cans or lipstick-stained wine glasses

haphazardly left on shelves all over his high-end market

by tipsy white women who don’t believe in crisis

until it hits their homes It was already hard not to bring

his work home But now it’s more dangerous,

this already thankless and unseen and ignorable work

It was too much even before all this impatience,

all this insistence, even before all this aggressive fear

made him miserable, visible, vulnerable, essential

Appendix 3: survey questions

1. Please rate your interest in the following subjects BEFORE the narrative medicine session.

a. Learning about the field of narrative medicine

    - Not at all interested

    - Somewhat interested

    - Moderately interested

    - Very interested

    - Extremely interested

b. Analyzing short pieces of literature 

     - Not at all interested

     - Somewhat interested

     - Moderately interested

     - Very interested

     - Extremely interested

c. Engaging in reflective writing

     - Not at all interested

     - Somewhat interested

     - Moderately interested

     - Very interested

     - Extremely interested

2. Please rate your level of confidence in the following skills BEFORE the narrative medicine 

session.

a. Listening to patients’ stories

     - Not at all confident

     - Somewhat confident

     - Moderately confident

     - Very confident

     - Extremely confident

b. Analyzing short pieces of literature

     - Not at all confident

     - Somewhat confident

     - Moderately confident

     - Very confident

     - Extremely confident

c. Engaging in reflective writing

     - Not at all confident

     - Somewhat confident

     - Moderately confident

     - Very confident

     - Extremely confident

3. Please rate your level of agreement with the following statements BEFORE the narrative medicine session.

a. Engaging in literary analysis and reflective writing can improve patient care.

     - Strongly disagree

     - Somewhat disagree

     - Neither agree nor disagree

     - Somewhat agree

     - Strongly agree

b. Engaging in literary analysis and reflective writing can reduce provider burnout.

     - Strongly disagree

     - Somewhat disagree

     - Neither agree nor disagree

     - Somewhat agree

     - Strongly agree

c. Engaging in literary analysis and reflective writing can improve connectedness to one’s colleagues.

     - Strongly disagree

     - Somewhat disagree

     - Neither agree nor disagree

     - Somewhat agree

     - Strongly agree

4. Please rate your interest in the following subjects AFTER the narrative medicine session.

a. Learning about the field of narrative medicine

     - Not at all interested

     - Somewhat interested

     - Moderately interested

     - Very interested

     - Extremely interested

b. Analyzing short pieces of literature

     - Not at all interested

     - Somewhat interested

     - Moderately interested

     - Very interested

     - Extremely interested

c. Engaging in reflective writing

     - Not at all interested

     - Somewhat interested

     - Moderately interested

     - Very interested

     - Extremely interested

5. Please rate your level of confidence in the following skills AFTER the narrative medicine session.

a. Listening to patients’ stories

     - Not at all confident

     - Somewhat confident

     - Moderately confident

     - Very confident

     - Extremely confident

b. Analyzing short pieces of literature

     - Not at all confident

     - Somewhat confident

     - Moderately confident

     - Very confident

     - Extremely confident

c. Engaging in reflective writing

     - Not at all confident

     - Somewhat confident

     - Moderately confident

     - Very confident

     - Extremely confident

6. Please rate your level of agreement with the following statements AFTER the narrative medicine session.

a. Engaging in literary analysis and reflective writing can improve patient care.

     - Strongly disagree

     - Somewhat disagree

     - Neither agree nor disagree

     - Somewhat agree

     - Strongly agree

b. Engaging in literary analysis and reflective writing can reduce provider burnout.

     - Strongly disagree

     - Somewhat disagree

     - Neither agree nor disagree

     - Somewhat agree

     - Strongly agree

c. Engaging in literary analysis and reflective writing can improve connectedness to one’s colleagues.

     - Strongly disagree

     - Somewhat disagree

     - Neither agree nor disagree

     - Somewhat agree

     - Strongly agree

7. Open-ended questions

a.  Through participation in this session, what (if anything) did you learn about yourself or your colleagues?

b. What (if anything) can you apply from this session to your clinical practice?

c. Please feel free to leave any additional comments about your experience in this session.
